# Incidence Trends of Atopic Dermatitis in Infancy and Early Childhood in a Nationwide Prescription Registry Study in Norway

**DOI:** 10.1001/jamanetworkopen.2018.4145

**Published:** 2018-11-02

**Authors:** Cathrine H. Mohn, Hege Salvesen Blix, Jon Anders Halvorsen, Per Nafstad, Morten Valberg, Per Lagerløv

**Affiliations:** 1 Department of General Practice, Institute of Health and Society, University of Oslo, Oslo, Norway; 2 Department of Pharmacoepidemiology, Norwegian Institute of Public Health, Oslo, Norway; 3 Department of Dermatology, Institute of Clinical Medicine, Oslo University Hospital, University of Oslo, Oslo, Norway; 4 Division of Mental and Physical Health, Norwegian Institute of Public Health, Oslo, Norway; 5 Department of Community Medicine and Global Health, Institute of Health and Society, University of Oslo, Oslo, Norway; 6 Oslo Centre for Biostatistics and Epidemiology, Oslo University Hospital and University of Oslo, Oslo, Norway

## Abstract

**Importance:**

With increasing prevalence of atopic dermatitis (AD) and its manifestation in most countries, together with the supporting evidence of the progression to other atopic phenotypes, AD has developed into a worldwide public health concern. The presence of the disease of has increased since the 1950s, but some recent studies suggest a stationary or decreasing trend.

**Objective:**

To analyze a nationwide health register based on prescription data to determine the incidence rate (IR) of AD in an entire pediatric population.

**Design, Setting, and Participants:**

All children resident in Norway younger than 6 years from January 1, 2009, through December 31, 2015, were included in this cohort study. Medical diagnoses and disease-specific medications were used as a proxy for identifying children with AD in this population-based prescription registry study. The prescription study was terminated in 2016. The total number of 295 286 disease-specific prescriptions was analyzed from August 2016 through December 2017. The hypothesis was formulated before, during, and after the data collection.

**Main Outcomes and Measures:**

All children with a medical diagnosis of AD or eczema based on at least 2 prescriptions of topical corticosteroids or at least 1 prescription of topical calcineurin inhibitors. Incidence rates per person-year (PY) and IR ratios were calculated.

**Results:**

A total of 295 286 disease-specific prescriptions were dispensed to 122 470 children, of whom 63 460 had AD and 56 009 (88.3%) had reimbursed prescriptions and associated AD diagnoses. The annual Norwegian study population (aged <6 years) increased from 357 451 children in 2009 to 373 954 in 2015. The overall IR increased from 0.028 per PY (95% CI, 0.028-0.029 per PY) in 2009 to 0.034 per PY (95% CI, 0.033-0.035 per PY) in 2014. For children younger than 1 year, the IR increased from 0.052 per PY (95% CI, 0.050-0.053 PY) in 2009 to 0.073 per PY (95% CI, 0.071-0.075 per PY) in 2014. In this age group, the IR was 53% higher in boys compared with girls (IR ratio, 1.53; 95% CI, 1.49-1.57; *P* < .001). The incidence proportion before the age of 6 years was 17.4% (95% CI, 17.2%-17.7%). The primary seasons for the onset of AD were winter and spring.

**Conclusions and Relevance:**

This nationwide study suggests an increase in the IR of pediatric AD, especially among children younger than 1 year. This study’s findings suggest that increase occurred with a higher IR during winter and spring seasons. Atopic dermatitis had an earlier onset in boys than in girls. During the study period, more than 1 in 6 children younger than 6 years had, at some point, been affected by AD.

## Key Points

**Question:**

Is the incidence rate of pediatric atopic dermatitis still increasing?

**Findings:**

In this cohort study, all children resident in Norway younger than 6 years from January 1, 2009, through December 31, 2015, were included. The overall incidence rate of atopic dermatitis increased from 0.028 per person-year in 2009 to 0.034 per person-year in 2014, and for children younger than 1 year, the incidence rate increased from 0.052 per person-year in 2009 to 0.073 per person-year in 2014.

**Meaning:**

This nationwide study suggests an increase in the incidence rate of pediatric atopic dermatitis, especially among children younger than 1 year.

## Teaser

This nationwide cohort study analyzes health register data from Norway to determine the incidence rate of atopic dermatitis among children younger than 6 years.

## Introduction

Atopic dermatitis (AD) is a common chronic, pruritic inflammatory skin condition affecting children and adults around the world. The disease episodically relapses and typically has an early onset, where approximately 80% develop the disease before 5 years of age.^1^ An increase in the occurrence of AD has been found in the Nordic countries since the 1950s.^2,3,4,5,6,7^ Although most of these studies have shown a growing trend, recent studies from Sweden and Denmark^3,8,9^ suggest that the frequency of small children with AD has stabilized or even decreased.

The pathogenesis of this chronic disease is heterogeneous, complex, and multifactorial.^10,11,12,13^ Inherited or acquired mutations in epidermal barrier proteins (such as filaggrin) are considered to be major drivers of change in the disease burden. Growing evidence suggests that environmental exposures play a key role in the pathogenesis of AD.^13,14,15^ The substantial variations in reported occurrences of AD between and within countries and between the seasons in temperate climate areas suggest that genetics alone cannot explain these variations.^8,16,17,18,19^ At present, a full understanding of how the seasons affect the occurrence of the disease is lacking.

Many scoring systems and diagnostic tools for AD diagnosis have been translated, validated, and used in numerous countries. In the absence of a uniform valid criterion standard test, an elevated risk of misclassification of the disease exists. Although several validated AD criteria are known for children and adults, the nomenclature associated with this (perhaps dichotomous^20^) disease is still under debate. The variety of existing clinical scoring systems, cross-sectional studies, and questionnaires (where the self-reporting of data can lead to recall bias) complicates the interpretation of the outcome, also preventing their comparison.^21^ Recently, national population-wide health registers containing prescription data have become available for research purposes, thereby providing a powerful epidemiologic research tool for identifying patients through dispensed prescriptions of disease-specific medication.^22,23,24,25,26^ The aim of this nationwide, retrospective register study was to examine the trends in the incidence rate (IR) of pediatric AD, using an algorithm for prescription data to determine the onset and seasonality in Norwegian children younger than 6 years.

## Methods

### Ethical Approval

This cohort study followed the Strengthening the Reporting of Observational Studies in Epidemiology (STROBE) guideline. The research study was approved in November 2015 by the Regional Committees for Medical and Health Research Ethics and the Norwegian Social Science Data Services, and both waived the need for informed consent because patient data were deidentified.

### Registers and Coding Classifications

The Norwegian Prescription Database (NorPD) monitors all prescribed medications that are dispensed in Norway, covering 5.2 million inhabitants (as of December 2015).^27^ Norwegian pharmacies are obliged to forward dispensed prescription data electronically to the NorPD. All dispensed prescriptions of topical corticosteroids (Anatomical Therapeutic Chemical Classification [ATC] code D07A) and the calcineurin inhibitors tacrolimus (ATC code D11AH01) and pimecrolimus (ATC code D11AH02) for external use were extracted from the NorPD database. The prescriptions were assigned with a pseudonym identification number, age, sex, month and year of birth and death, dispensing date, generic medication name, and ATC codes.^28,29^ Only the reimbursed prescriptions had associated codes from *International Statistical Classification of Diseases and Related Health Problems, Tenth Revision *(*ICD-10*), and *International Classification of Primary Care, Version 2* (*ICPC-2*). The reimbursed prescriptions are primarily prescribed for chronic illnesses and require a minimum of 3 months’ use annually.^30^ The population statistics were obtained from Statistics Norway.^27^

### Study Population

All children resident in Norway aged 0 to 6 years from January 1, 2009, through December 31, 2015, were included. The annual study population (aged <6 years) increased from 357 451 children in 2009 to 373 954 in 2015. Children who were dispensed AD-specific medication (topical corticosteroids or calcineurin inhibitors or both for external use) were investigated to determine whether they had AD. Children who were dispensed AD-specific medication before December 31, 2008, were excluded from this study.

### Algorithm for Identifying Children With AD

Children were considered to have AD if they met at least 1 requirement for criterion 1 or 2. Criterion 1, based on a medical diagnosis, included children with recorded reimbursement prescriptions containing associated disease-specific diagnoses of AD or eczema recorded as *ICD-10* code L20 or *ICPC-2* code S87. Criterion 2, based on dispensed disease-specific medication, included children with nonreimbursement prescriptions (not containing an AD diagnosis as in criterion 1). The child was considered to have AD if he or she, within 1 year, had at least 2 prescriptions of topical corticosteroids or at least 1 prescription of topical calcineurin inhibitors.

Children classified by criterion 2 with co-occurring *ICD-10* or *ICPC-2* codes for skin diagnoses (which might lead to identical treatments) or co-occurring skin disease–specific medications (primarily prescribed for other diseases) were not considered to have AD. eMethods in the Supplement provides further explanations of the algorithm used. According to the requirement of more than 2 prescriptions of topical corticosteroids within 1 year (criterion 2), the IR based on dispensed disease-specific medication for 2015 could not be calculated.

### Statistical Analysis

The data were analyzed from August 2016 through December 2017 using Stata/MP software (version 14.2; StataCorp LLP). We used the Poisson regression procedure to calculate the IR per person-year (PY) and incidence rate ratios (IRRs) with 95% CIs. Differences between the IRs were tested by χ^2^ tests. *P* < .05 (2-sided test) was deemed statistically significant. We calculated the IRs according to sex, age, calendar year, and their interactions. To determine the trends over time, 2009 was set as the reference year. Incidence proportion (cumulative incidence) of AD onset was estimated as the proportion of children in the population who, based on the algorithm, ever had AD using the Kaplan-Meier method.^31^

In a separate analysis, we used the Poisson regression procedure to investigate the seasonal variations in the IRs for AD. The seasons were defined as spring (March-May), summer (June-August), autumn (September-November), and winter (December-February).

## Results

### Prescription and Patient Selection

A total of 295 286 disease-specific prescriptions were dispensed to 122 470 children. Of these, 63 460 children had AD according to the algorithm. Furthermore, 56 009 of these children (88.3%) had been provided by physician with reimbursed prescriptions and associated AD diagnoses (criterion 1).

### Trends in IR of AD and Incidence Proportion

**Figure 1.**
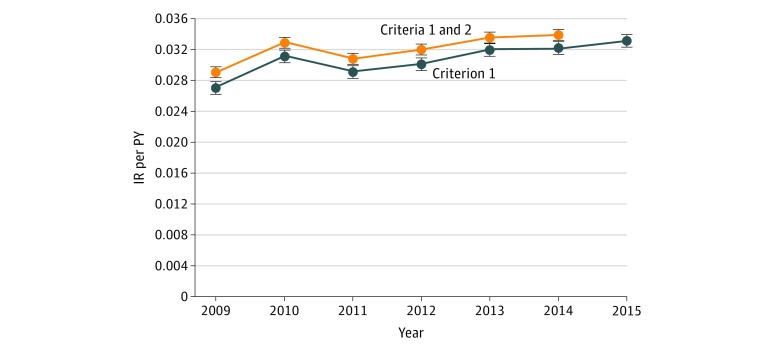
Incidence Rate (IR) per Person-Year (PY) of Atopic Dermatitis During the First 6 Years of Life Data are from the Norwegian Prescription Database from January 1, 2009, through December 31, 2015. Error bars indicate 95% CI. The upper curve displays the IR per PY determined by reimbursed (criterion 1) and nonreimbursed (criterion 2) medications as proxies for atopic dermatitis; the lower curve (year 2009-2015) displays the IR for reimbursed medication only (criterion 1).

The IR for the children with AD showed that, excluding 2010, a steady increase occurred throughout the study period. The IR increased from 0.028 per PY (95% CI, 0.028-0.029 per PY) in 2009 to 0.034 per PY (95% CI, 0.033-0.035 per PY) in 2014, which represents an increase of 16.8% (IRR, 1.17; 95% CI, 1.14–1.20; *P* < .001) (Figure 1 and eTable 1 in the Supplement).

**Figure 2.**
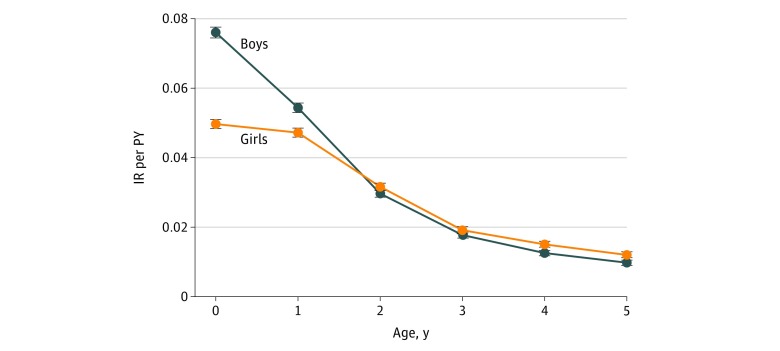
The Incidence Rate (IR) per Person-Year (PY) in Children With Atopic Dermatitis According to the Indicated Age Group Data are from the Norwegian Prescription Database from January 1, 2009, through December 31, 2015. Error bars indicate 95% CI.

The increasing trend displayed in Figure 1 was mainly attributable to children younger than 1 year (Figure 2 and eTable 2 in the Supplement). The IR in this specific age group increased from 0.052 per PY (95% CI, 0.050-0.053 per PY) in 2009 to 0.073 per PY (95% CI, 0.071-0.075 per PY) in 2014, which corresponds to an increase of 42.0% (IRR, 1.42; 95% CI, 1.32–1.53; *P* < .001).

During the first year of life, the IRs of boys increased from 0.063 per PY (95% CI, 0.061-0.066 per PY) in 2009 to 0.088 per PY (95% CI, 0.085-0.092 per PY) in 2014, representing an increase of 41% (IRR, 1.41; 95% CI, 1.33-1.49; *P* < .001). In comparison, the IRs in girls in the same age group ranged from 0.040 per PY (95% CI, 0.038-0.043 per PY) in 2009 to 0.057 per PY (95% CI, 0.055-0.060 per PY) in 2014, representing an increase of 42% (IRR, 1.42; 95% CI, 1.32-1.53; *P* < .001). The IRs in the remaining age groups (>1 year) were considered to be stable (Figure 2).

**Figure 3.**
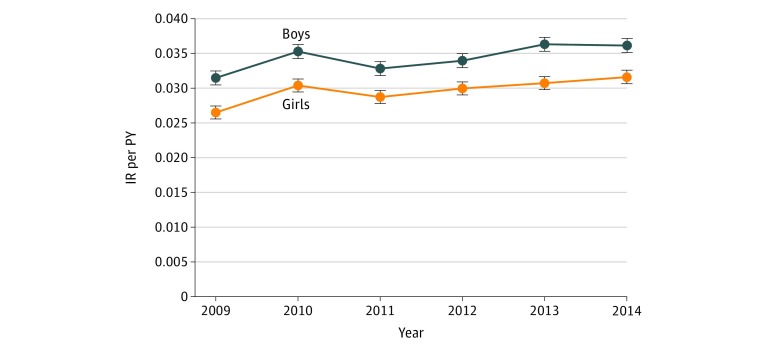
The Interaction of the Incidence Rate (IR) per Person-Year (PY) of Atopic Dermatitis Between Age and Sex Includes boys and girls younger than 6 years. Data are from the Norwegian Prescription Database from January 1, 2009, through December 31, 2014. Error bars indicate 95% CI.

We found an interaction between age and sex, with boys having a higher IR compared with girls, especially those younger than 1 year (Figure 3). During the first year of life, the IR for boys was 0.076 per PY (95% CI, 0.075-0.077 per PY) compared with 0.050 per PY for girls (95% CI, 0.049-0.051 per PY). In this age group, boys thus had a 53% (IRR, 1.53; 95% CI, 1.49-1.57; *P* < .001) higher IR than girls (Table). During the second year of life, the overall IRs for boys and girls were 0.054 per PY (95% CI, 0.053-0.056 per PY) and 0.047 per PY (95% CI, 0.046-0.048 per PY), respectively. In this age group, boys had a 15% (IRR, 1.15; 95% CI, 1.12-1.19; *P* < .001) higher IR compared with girls. After 2 years of age, the IRs of AD were considered as equal for both sexes (Figure 3).

**Table.** Incidence Proportion During the Years 2009-2014^a^

Age, y	No. of AD Events	Incidence Proportion (95% CI), %
0	22 285	6.30 (6.22-6.39)
1	39 548	11.06 (10.91-11.21)
2	49 695	13.79 (13.59-13.98)
3	55 675	15.37 (15.14-15.61)
4	60 064	16.54 (16.27-16.80)
5	63 460	17.44 (17.15-17.74)

Abbreviations: AD, atopic dermatitis.

^a^Data are given as the incidence proportion of children (percentage) with AD during the years 2009 through 2014.

**Figure 4.**
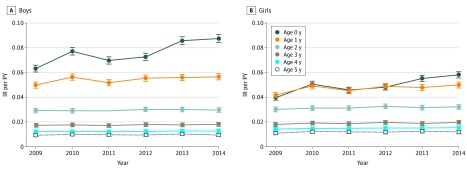
The Annual Incidence Rate (IR) per Person-Year (PY) of Atopic Dermatitis for Boys and Girls Includes children younger than 6 years. Data are from the Norwegian Prescription Database from January 1, 2009, through December 31, 2014. Error bars indicate 95% CI.

The IRR of boys compared with girls varied between years, ranging from a maximum 19% (IRR, 1.19; 95% CI, 1.14-1.24; *P* < .001) higher IR in boys in 2009 to the minimum 13% (IRR, 1.13; 95% CI, 1.11-1.21; *P* < .001) in 2012 (Figure 4). The IR of AD for both sexes was highest during the first year of life, ranging from 0.063 per PY (95% CI, 0.062-0.064 per PY) at younger than 1 year to 0.011 per PY (95% CI, 0.010-0.011 per PY) at younger than 5 years. Overall, during the 6 years of the study period, the incidence proportion of AD among those younger than 6 years was 17.4% (95% CI, 17.2%-17.7%) (Table).

### Seasonality

The seasonal debut (seasonal IR) of AD peaked during winter and spring. The seasonal IRs for Norwegian children with AD were 0.036 per PY (95% CI, 0.036-0.037 per PY [n = 17 778]) in the winter and 0.038 per PY (95% CI, 0.038-0.039 per PY [n = 18 835]) in the spring. The lowest seasonal IR occurred in the summer at 0.026 per PY (95% CI, 0.025-0.026 per PY [n = 12 475]) and the second lowest in the autumn at 0.030 per PY (95% CI, 0.029-0.030 per PY [n = 14 372]). Thus, the seasonal IR in the spring was 49.6% higher (IRR, 1.50; 95% CI, 1.46-1.53; *P* < .001) than in the summer (reference category). The IRRs for autumn and winter were 1.16 (95% CI, 1.13-1.19) and 1.41 (95% CI, 1.38-1.45), respectively.

## Discussion

This nationwide study, based on disease-specific dispensed prescriptions, displays an increase in the IR of AD, especially among children younger than 1 year. More than 1 in 6 children younger than 6 years had, at some point during the study period, been affected by AD. To convey the true magnitude of disease risk and disease onset in the study population, we presented population-based incidence estimates regarding AD. However, to date only a few nationwide studies report IRs for AD in children. What, to our knowledge, is the only globally and uniform validated research study of AD, the International Study of Asthma and Allergies in Childhood (ISAAC), showed that more than 20% of children are affected by AD in some countries.^32^ The latest available data (phase 3) revealed that AD continues to increase worldwide, especially in young children (ages 6-7 years).^32^

Although the ISAAC study found an increasing trend of small children with AD, a 2015 register study of the IRs of AD in Denmark and Sweden by Henriksen et al^8^ concluded that the number of children with AD had stabilized during the same period. In contrast to our study, the report of Henriksen et al^8^ only applied *ICD-10* diagnoses used in hospital settings to examine the trends in the IR of AD. Most patients with AD, however, only occasionally require hospital or specialist therapy. Consequently, the diagnoses in primary care (*ICPC-2*) should correspond better to the general population as a less restricted group, compared with those in hospitals.

Three cross-sectional questionnaire studies of AD among Norwegian schoolchildren found self-reported prevalence rates of 13.4%, 21.1%, and 20.8% in 1985, 1995, and 2000, respectively.^4,5^ The analysis included children aged 9 to 11 years with questionnaires that sought to determine whether the child had ever displayed symptoms of AD. Despite the obvious methodologic differences, these findings are compatible with our data showing that 17.4% of the children were affected by AD before reaching the age of 6 years.

Our results showed that AD had an earlier onset in boys than in girls. After the age of 2 years, the sex differences were leveled out. The results from a Danish birth cohort study describe similar findings in infants to the age of 18 months.^33^ Sex differences among children diagnosed with asthma is a recognized phenomenon, but large-scale longitudinal studies of age-related sex differences in young children with AD are limited.^33,34,35,36^ Skin hydration, surface pH, and sebum content have repeatedly been observed in infants according to sex and age without consistent findings.^37,38,39^ However, the temperament of infants may differ between sexes.^40,41^ An experimental study of a group of US infants^42^ showed that boys appear to have a more limited capacity for self-regulation compared with girls at the same age. Theoretically, if boys with AD express more discomfort compared with girls, boys would be more likely to visit a physician at a younger age than girls.

Our study showed an increasing IR during a short time span, which may be a consequence of a change in exposure to environmental factors. Two previous observational studies from Finland and Germany of children with AD and allergies and living in geographically adjacent areas (who were genetically related)^43,44^ have shown that the environment rather than a genetic predisposition could have aggravated the condition. An alteration in genetically predisposed individuals may not be ruled out, but in the German study^43^ and the present study, these effects are conceivably less relevant because the increase in the IR occurred during a short period. Moreover, the Norwegian, Danish, and Swedish people are genetically related, which means that the environment, together with disparities in design and methods, could have caused the divergent findings in the present study compared with the study of Henriksen et al.^8^

In the present study, seasonal variations in the IR of AD showed a distinct peak during winter and spring seasons, a result of which has previously been shown by other researchers.^8,13,45,46^ Former studies have demonstrated the association between the onset of pollination and the manifestation of AD and pruritus.^15,17,18,19^ Although airborne pollen may contribute to the identified springtime peak, as yet no clear explanations exist for the seasonal peak in the IR for AD during the winter season. According to a recent experimental study, infants’ stratum corneum water content indicated a significant seasonal difference (higher in early summer than in autumn).^47^ One may speculate whether the water content is affected by the ambient temperature and has relevance for the skin barrier. Our results suggest that environmental conditions associated with season may play a role in the onset and perhaps worsening of the disease.

Our results showed a higher IR of AD in 2010. According to the climate statistics, the winter of 2010 in Norway and Sweden was extraordinarily cold.^48^ The Norwegian sales of topical corticosteroids and glucocorticoid inhalers for children, the IR for AD in the present study (Figure 1), and the IR of asthma in Sweden peaked in the same year.^8,49^ The ISAAC study^45^ found that lower latitudes and eastern longitudes were slightly but significantly associated with higher prevalence of current symptoms of AD, globally and in all age groups. Our hypothetical speculation, however, is merely based on an ecological comparison of geoclimatic factors, and more studies are required to understand the seasonal variations in this complex and heterogeneous skin disease.

### Strengths and Limitations

The main strength of this study is the large volume of data, which allowed us to examine the IRs of AD for the entire Norwegian child population younger than 6 years, thereby obtaining reliable results with high significance. The Norwegian child welfare remained practically unchanged during this short study period. However, whether improved awareness of AD, increased urbanization or access to health care facilities, changes in consultation practice, increased environmental exposures, or genetic alterations contributed to the increasing trend found in the NorPD database is a question with important public health implications. Most likely a combination of many relevant causal processes together with an actual increase of children with AD are responsible.

The identification number on the dispensed prescription was missing in 4.5% of all the received prescriptions from the NorPD. These prescriptions were excluded from the present study.^29^ However, AD is defined as a chronic illness. Hence, a child with AD likely would have received prior or subsequent (or both) medical treatment. Thus, we suspect that the excluded prescriptions belong to children already included in this study. Our results should accordingly be robust regarding the missing prescriptions.

Minor episodes of AD may have gone undetected in our study. In Scandinavian countries, the mildest topical corticosteroids can be obtained over the counter. However, Norwegian children with chronic illnesses (including AD) have the right to receive reimbursed prescriptions, which provides the child with necessary medical treatments and medications free of charge. In comparison, purchase of over the counter medications vs reimbursed medication stands out as a more expensive alternative.^30,50^ Moreover, the diagnoses assessed based on reimbursement prescriptions (criterion 1) are more likely to reflect children with higher disease burden, which may have led to fewer diagnostic errors. The reimbursed prescriptions additionally provided this study with a high probability to have covered children with AD from all socioeconomic strata.

Topical corticosteroids are frequently prescribed for a relatively broad group of skin disorders, which may lead to uncertainty about the validity of the medication proxies used to identify children with AD.^22,23,24^ The algorithm used in the present study was primarily based on physician-given diagnoses (criterion 1 [88.3%]), and the use of medication proxy (criterion 2) was minimized to only 11.7% of the participants considered. Mulder et al^23^ proposed that 2 or more annual prescriptions of topical corticosteroids yield a sensitivity value of only 40% and a positive predictive value of 60%.^24^ Compared with the proposed values, our algorithm covered a higher fraction of the children with AD, providing us with a higher sensitivity value. The set of non-AD criteria (criterion 3) additionally increased the positive predictive value.

## Conclusions

The present nationwide study outlines a recent longitudinal increase in the IR of pediatric AD, mainly among children younger than 1 year. This increase has occurred during a short period and is possibly related to environmental and lifestyle factors in genetically predisposed individuals. Atopic dermatitis is more common among boys than girls at an early age, and with a higher IR during the winter and spring seasons.

## Notes

Supplement. **eMethods.** Algorithm for Defining Children With Atopic Dermatitis (AD) **eTable 1.** Incidence Rates (IR) and Incidence Rate Ratio (IRR) per Person-Year for Atopic Dermatitis (AD) **eTable 2**. The Number of Events and IRs per Person-Year for Boys and Girls With AD by Age
