# Errors in Figure Titles

**DOI:** 10.1001/jamanetworkopen.2023.6504

**Published:** 2023-03-14

In the Original Investigation titled “Incidence Trends of Atopic Dermatitis in Infancy and Early Childhood in a Nationwide Prescription Registry Study in Norway,”^1^ published November 2, 2018, there were errors in the figure titles. The title of Figure 2 should have been “Interaction of the Incidence Rate (IR) per Person-Year (PY) of Atopic Dermatitis Between Age and Sex,” and the title of Figure 3 should have been “Incidence Rate (IR) per Person-Year (PY) in Children with Atopic Dermatitis According to the Indicated Age Group.” This article has been corrected.^1^

## References

[1] Mohn CH, Blix HS, Halvorsen JA, Nafstad P, Valberg M, Lagerløv P. Incidence trends of atopic dermatitis in infancy and early childhood in a nationwide prescription registry study in Norway. JAMA Netw Open. 2018;1(7):e184145. doi:10.1001/jamanetworkopen.2018.4145 30646341 PMC6324394

