# Supplementary material for: Incidence Trends of Atopic Dermatitis in Infancy and Early Childhood in a Nationwide Prescription Registry Study in Norway
Source: JAMA Netw Open. 2018 Nov 2;1(7):e184145. doi: 10.1001/jamanetworkopen.2018.4145 (PMC6324394; doi:10.1001/jamanetworkopen.2018.4145)
Supplement: Supplement. — eMethods. Algorithm for Defining Children With Atopic Dermatitis (AD) eTable 1. Incidence Rates (IR) and Incidence Rate Ratio (IRR) per Person-Year for Atopic Dermatitis (AD) eTable 2. The Number of Events and IRs per Person-Year for Boys and Girls With AD by Age [file jamanetwopen-1-e184145-s001.pdf]

Supplementary Online Content

Mohn CH, Blix HS, Halvorsen JA, Nafstad P, Valberg M, Lagerløv P. Incidence trends of atopic dermatitis in infancy and early childhood in a nationwide prescription registry study in Norway. *JAMA Netw Open*. 2018:1(7):e184145. doi:10.1001/jamanetworkopen.2018.4145

eMethods. Algorithm for Defining Children With Atopic Dermatitis (AD)
eTable 1. Incidence Rates (IR) and Incidence Rate Ratio (IRR) per Person-Year for Atopic Dermatitis (AD)
eTable 2. The Number of Events and IRs per Person-Year for Boys and Girls With AD by Age

This supplementary material has been provided by the authors to give readers additional information about their work.

© 2018 Mohn CH et al. *JAMA Network Open.*

eMethods. Algorithm for Defining Children With Atopic Dermatitis (AD)

Children were considered to have AD if they met at least one requirement for either Criteria 1 or 2.

Criterion 1 - based on ICD-10 or ICPC-2 diagnoses:

- ≥ 1 hospital or specialist (dermatologist) contact for: L20 “atopic dermatitis”
- ≥ 1 GP contact for: S87 “dermatitis/atopic dermatitis”

Criterion 2 - based on disease-specific medication (ATC-codes):

- ≥ 1 dispensed prescription for: D11AH “agents for dermatitis: tacrolimus or pimecrolimus” [*1]
- ≥ 2 dispensed prescriptions for: D07 “corticosteroids for topical use” within 12 months **without any of the following non-AD Criteria.**

NON-AD CRITERIA

Children with co-occurring medical skin diagnoses (that might lead to identical treatment) or with co-occurring disease-specific medication (primarily prescribed for other diseases) were not considered to have AD by the following non-AD Criteria.

Co-occurring skin diagnoses (based on ICD-10 or ICPC-2):

≥ 1 diagnosis of either:

- **ICD-10**: L21 “seborrhoeic dermatitis,” L22 “diaper dermatitis” L23 “allergic contact dermatitis,” L24 “irritant contact dermatitis,” L25 “unspecified contact dermatitis,” L26 “exfoliative dermatitis,” L27 “dermatitis due to substances taken internally,” L28 “lichen simplex chronicus and prurigo,” L30 “other dermatitis,” L40–L45 “papulosquamous disorders,” L53 “other erythematosus conditions,” L55 “sunburn,” L56 “other acute skin changes due to ultraviolet radiation,” L80 “vitiligo,” L90 “atrophic disorders of the skin,” L93 “lupus erythematosus” OR
  - **ICPC-2**: S86 “seborrhoeic dermatitis,” S88 “dermatitis contact/allergic,” S89 “diaper dermatitis” L88 “allergic contact dermatitis,” S80 “solar keratosis/sunburn,” S82 “exfoliative dermatitis,” S06 “rash localized,” S07 “rash generalized,” S08 “skin color change,” S91” psoriasis,” S99 “skin diseases” OR

© 2018 Mohn CH et al. *JAMA Network Open.*

Co-occurring disease-specific medication (based on ATC):

≥ 1 dispensed prescription for either:

- D05 “antipsoriasics” or D02AF “salicylates for dermatological use” or D07AD “corticosteroids (group IV) including clobetasol” [*2]

[*1] *Calcineurin inhibitor crème/ointment 0.03% is indicated for adults, adolescents, and children from 2 years of age and are only prescribed for moderate-to-severe AD.* [1E]

[*2] *For prescriptions of corticosteroid group IV (without ever having a diagnosis of AD). AD is not treated singly with group IV.*

eREFERENCES

1E. AS F. Felleskatalogen Oslo 2017 [17.03.17]. Available from: https://www.felleskatalogen.no/medisin/protopic-leo-563076.

© 2018 Mohn CH et al. *JAMA Network Open.*

eTable 1. Incidence Rates (IR) and Incidence Rate Ratio (IRR) per Person-Year for Atopic Dermatitis (AD)

Annual IR and IRR per person-year of children with AD (with 95% CIs)				
Year	IR	95% CI	IRR	95% CI
2009	0.029	0.028 - 0.030	1.00	Reference
2010	0.033	0.032 - 0.033	1.13	1.10 - 1.16
2011	0.031	0.030 - 0.031	1.06	1.03 - 1.09
2012	0.032	0.031 - 0.033	1.10	1.07 - 1.13
2013	0.034	0.033 - 0.034	1.16	1.13 - 1.19
2014	0.034	0.033 - 0.035	1.17	1.14 - 1.20

Data show he relatively steady increase (excluding 2010) during the study period (Figure 1) presented with 95% CIs.

© 2018 Mohn CH et al. *JAMA Network Open.*

eTable 2. The Number of Events and IRs per Person-Year for Boys and Girls With AD by Age

IR per person-year of children with AD, by age and gender					
Age	Boys	IR for Boys	Girls	IR for Girls	Total
0	13693	0.076	8592	0.050	22285
1	9352	0.054	7911	0.047	17263
2	4975	0.030	5172	0.032	10147
3	2912	0.018	3068	0.019	5980
4	2025	0.013	2364	0.015	4389
5	1546	0.010	1850	0.012	3396
Total	34503		28957		63460

Data are shown in Figure 2.

© 2018 Mohn CH et al. *JAMA Network Open.*
